# The Social Science Transactions

**Published:** 1858-07

**Authors:** 


### THE SOCIAL SCIENCE TRANSACTIONS.

THE formation, last year, of a society for the study and cultivation of social science, formed an important epoch in our national history. The society met at Birmingham, under the presidency of Lord Brougham, and supported by many of the wisest and best of the land, it passed off with all that success that could be expected from it, and with even more success than was anticipated even by its founders. The department of public health, presided over by Lord Stanley, possessed to us most interest. The Transactions of the society, now in our hands, indicate how ably this department was sustained.

The sanitary papers before us are too numerous to admit of complete analysis, and too compact to allow of condensation. Instead, therefore, of giving either analysis or epitome, we select for our readers, from certain of these papers, such parts as we think will afford them most information. The admirable address of Lord Stanley has been commented on in the present number, in the report of the council of the Epidemiological Society.

*Districts and Death-Rates.*—Dr. Headlam Greenhow, in a paper on "The Analytical Study of the Statistics of Public Health," supplies the following:—

"There are districts in England and Wales which, in point of salubrity, occupy every possible intermediate position between the unhealthiness of Liverpool and the healthfulness of the rural districts with which it has here been contrasted. As a general rule, large towns commonly present a high death-rate. The public health of smaller towns and of rural places is, for the most part, comparatively good. This remarkable difference between the state of the public health in different districts—a difference that is rarely caused by the climate, position, or natural character of the places—has been usually, and I believe correctly, ascribed to adventitious circumstances incidental to human occupation, and especially to the rural or urban character of the districts. This general inference has been made the basis of all arguments in favour of employing measures for the amelioration of the public health of towns, the insalubrity of which has been, perhaps too hastily and exclusively, attributed to a few only of the most prominent evils to which urban populations are exposed.

"The diseases by which the larger mortality of unhealthy districts is occasioned, have also been hastily inferred from data equally incomplete. The diseases to which the term 'zymotic' is applied are usually believed to be the chief causes of the excess of deaths in unhealthy districts; and the term 'zymotic disease' is often, and without qualification, employed almost as if it were synonymous with preventable disease. The incorrectness of such language may be well shown by a single illustration. The term 'zymotic' is, perhaps, most correctly applied to those diseases which, being produced by a special poison, are capable of propagation by contagion. The infinitesimal drop of smallpox lymph, or the inappreciable contagion of scarlet-fever, is not only capable of exciting these diseases, but sets up an action in the body which may well be considered as a kind of fermentation, since one of its results is the formation of a similar poison capable of exciting the special disease, but in a quantity immensely greater than the amount originally received. However we may speculate upon the original cause of these and analogous diseases, we are now only acquainted with them as the consequence of being brought into immediate relation with a poison generated in the bodies of the sick. As has been alleged of certain diseases of cattle, they may have originated among a particular race of men exposed to peculiar conditions, and spreading from this origin—the conditions necessary to their spontaneous production having ceased—they are now propagated by means of contagion. Certain it is, that we are unacquainted with any other mode of preventing them than the exclusion of their special poison. The admission of any of these poisons to a person unprotected by a previous attack of the disease—for it is a common quality of these diseases that one attack very greatly diminishes the liability to a second—rarely fails to induce the disease, whatever the sanitary condition of the district in which he may reside. Again, how can we hope to prevent certain epidemic diseases, as dysentery, influenza, and fever, which evidently depend upon causes that, generally diffused over infected districts, act upon large numbers of persons, irrespective of residence or of sanitary condition? It is, indeed, true of several of these diseases, that the adoption of precautions, and particularly of such as are calculated to maintain the purity of the atmosphere, will render the dwellers in particular districts less liable to suffer severely from the epidemic influence. Thus, whilst the *amount* of the disease may be equal in salubrious and insalubrious places, its *intensity* may so greatly vary that the same epidemic which causes but little inconvenience or danger in the former, may prove of pestilential severity in the latter. Visitations of epidemic disease are, however, only of occasional occurrence. However formidable may be their ravages at the time, when the mortality occasioned by them is subdivided over a series of years it adds but little to the average general death-rate, which, nevertheless, continues chronically high in some places, and permanently low in others." (p. 368-9.)

*Sewerage of Towns.*—Mr. R. Rawlinson, in a paper on the above subject, gives the subjoined conclusions:—

"That natural streams down valley lines should never be converted into sewers.

"That all valley lines should be improved to admit of the escape of surface water in a natural manner.

"That sewers should be graduated so as to remove roof, yard, street, soil, and subsoil water from the town.

"That house-drains should remove all subsoil, surface, and slop-water from houses into the sewers.

"That all sewers should have flood-water overflows connected with valley culverts, so as to lead such surplus into rivers or streams direct or for filtration and disinfection.

"That all sewers should be fully ventilated. That there should be easy means of inspecting and full means for flushing, cleansing, and repairing.

"That where new works have to be set out neither sewers nor drains should enter nor pass between inhabited buildings, and that there should be full means for external ventilation at the upper ends of all drains, outside the walls of buildings. Any sewers or drains existing under houses should be air-tight and fully ventilated, so as to prevent a possibility of internal gaseous contamination to such house or houses.

"That sewers should not join at right angles. But that tributary sewers should deliver sewage in the direction of the main flow at an angle never greater than thirty degrees.

"That sewers at all curves, or change of direction, should have extra fall to compensate for increased friction.

"That all sewers and drains should be laid in straight lines and at true gradients from point to point. That at each change of line or gradient there should be means of inspection and of cleansing provided by manholes and lampholes.

"That sewers should never join at the same level, but that the tributary sewer should have increased fall at the junction with the main sewer.

"That all junctions of branch sewers and house drains should be above the ordinary level of sewage flow in the main sewer, and that branch sewers and house drains should have extra fall before and at the point of junction.

"Earthenware pipes above twelve inches in diameter need not be used, as, in general, they are more expensive than brick sewers one-half larger in sectional area.

"All bricks for sewers should be moulded to the radii required. They should be hard and vitreous, free from lime, and such as will endure great wear.

"The best hydraulic mortar should be made and used fresh, so as to produce sound and lasting work.

"Earthenware pipes of equal diameters should not be joined, but the lesser should join the greater, and there should be extra fall at the junction.

"The inlets to all pipe drains should be guarded by a trap or by means which will not allow any substance of diameter equal to the full bore of the pipe to be inserted.

"At all manholes there should be means of flushing, as also in all flat districts and at the upper ends of branch sewers and tributary drains.

"Sewers and drains act as land drains to the full depth to which they are laid. In wet subsoils this should be considered in settling the sectional dimensions of sewers and drains.

"Sewage may be discharged into rivers, and into the sea, without injury to health, if proper means are used to dilute or to filter and disinfect the fluid refuse.

"Sewage, under certain conditions, may be applied to land for agricultural use, so as to be the cheapest mode of disposing of the same.

"Large volumes of sewage should not be concentrated and poured out at any one point. Several points of discharge, as wide apart as possible, will allow of fuller dilution.

"A duplicate system of sewers cannot be necessary under any circumstances. If there are natural streams they must not be used as sewers.

"It may be stated generally that town sewerage is practicable. That is, sewers which will perform the required work can be executed, and at a cost within the means of the poorest body of ratepayers." (pp. 43-45.)

*Hygiene and Military Education.*—Sir John Richardson, speaking on this subject, says:—

"It might be expected that a people whose humanity is vaunted, whose knowledge of science is equal to that of any other nation, and whose means are inexhaustible, would at home, at least, and in our colonies, take care that the lives of our defenders should not be wantonly thrown away by placing barracks in the focus of disease, or neglecting to provide the necessary appliances for cleanliness and sufficient ventilation and drainage. Even the money value of the men annually sacrificed by this neglect forms no inconsiderable item in the national expenditure, and ought long ago to have incited the war authorities to invoke the aid of science for the mitigation of this evil. But what is the actual state of the case? Take our West Indian Colonies, for instance, and it will be found that the rule has been to place the barracks in situations unusually exposed to malaria. St. Vincent and Demerara are, I believe, the only exceptions, and in one of these the construction of the buildings is unsuited to the climate. At Barbadoes, the iron barracks, were contrary to the advice of the chief medical officer, erected in the lower part of the Savannah, near the burying-ground, and on outbreaks of yellow-fever it has repeatedly been found necessary to move the troops to an encampment in a higher and more airy and healthier locality. At home, many of our barracks and public hospitals are badly ventilated, ill-provided with the means of preserving the comforts and decencies of civilization, deficient in a supply of good water, situated near the deposits of the excrementitious matters of large towns, and surrounded by narrow lanes of the lowest kind of houses, the dwellings of the meanest and filthest members of the community. These evils are in part owing to a misplaced parsimony in the Government declining to allot funds for the purchase of the neighbouring grounds when a site is selected for an hospital or barrack. In consequence of this neglect, no sooner is a barrack built, than the vicinity is covered with slightly-built houses for lodging the families of the married soldiers, at a rent of one shilling or eighteen pence a week for each room, conveniences and drainage being the last things thought of. Low pot-houses also arise, and the classes that prey on the soldier and sailor crowd into the district, where their stews prove unfailing nurseries of contagious disease. Such places are still in existence in our garrison and seaport towns, though some have been recently reduced at a great cost, which might have been got in the first instance for a small sum. Had the engineers who planned and executed the public buildings to which I allude been conversant with the laws of sanitary science, they would scarcely have failed to urge a due attention to them upon the heads of the departments." (p. 450.)

*Air-Tight Coffins.*—Mr. J. J. Salt recommends for burial purposes an air-tight coffin, which is thus described:—

"The improved coffin referred to is made in the usual form and external appearance; the material of which it is composed is sheet zinc, strengthened at the edges by a bead of the same material; this bead is flattened at the top, and thus forms a broad substantial bearing for the lid, which is merely laid in its place and soldered to the flat surface of the bead.

"Besides its cheapness, this coffin possesses the further advantage of being lighter than the ordinary wooden one.

"The durability of sheet zinc is well known; it arises from the formation of a film of oxide on the surface, which is so compact and uniform as to serve as a kind of protecting varnish to the metal beneath. Unless exposed to mechanical violence, or the action of corrosive acids capable of dissolving this oxide, the durability of zinc is so great, that there is every reason to believe that a zinc coffin, buried at a moderate depth in the earth, will last beyond the time required for the elements of the body to arrange themselves in such a manner as not to be liable to any further decomposition.

"It is the opinion of several practical chemists, of whom I have made inquiries, that a body hermetically sealed in such a coffin would not go through the ordinary course of putrefaction, as such decomposition consists chiefly of the combination of the materials of the body with the oxygen of the air; and that this decomposition takes place on account of the feeble affinities by which the elements forming the soft tissues and the fluids of the body are held together. When the small quantity of air enclosed in the coffin has given up its oxygen, this action could go no further, and changes of a different nature would take place; and the loosened elements of the body would, under these circumstances, arrange and re-arrange themselves into various compounds, which would go on changing until they at last formed substances such as adipocire, in which the elements are held together by more powerful affinities; that further chemical change would then cease, and such compounds, when once formed, would not be liable to dangerous decomposition by mere exposure to the atmosphere.

"Besides the safety this coffin ensures after burial, it will be of the greatest value in preventing the mischievous consequences that so frequently result from the decay of the body between the time of death and interment.

"Such coffins may be made of any size to order in a few hours, and the body soldered up in its air-tight case within eight or ten hours after death." (p. 455-6.)

*On Central or Local Action.*—In defence of the principle of central action in matters of local improvement, Mr. Tom Taylor argues as follows:—

"Reviewing what has been said, it will be found that the necessity of central action on matters of local improvement is maintained,—

"1. To confer powers for such improvement cheaply and effectually; to invest with the legal character of 'towns' areas of dense population not having yet acquired a known and defined boundary; and to fuse into a consistent whole existing local Acts, and a general measure of town improvement.

"2. When such powers are conferred, to forward generally the wise and efficient exercise of them by diffusing the light of a general experience, and by communicating the results of such special inquiries as the central department may be charged to make, by advising in cases of doubt or difficulty, and generally by assisting, but never superseding, local efforts.

"3. To protect posterity, by examining and deciding upon application for leave to mortgage rates.

"4. To report to Parliament on the exercise of local powers.

"5. To act as a court of appeal against local oppression in certain specified cases, and a court of *mandamus* in cases of local default.

"I have spoken of town improvement generally, for I conceive it impossible to pick out any particular functions of the town-improving authority which can be said peculiarly to concern the public health.

"Improvement in the health of a town population is the best evidence that the town is well governed.

"Neither have I made any attempt to enumerate the other duties distinct from those directly relating to town improvement, which a central department may perform for general enlightenment on hygienic questions, and to forward the infant science of preventive medicine.

"In conclusion, I would wish very distinctly to reiterate the opinion, that local improvement is primarily and specially a local duty; that the central authority, which should attempt to supersede the fulfilment of this local duty by local agency, instead of aiding, enlightening, and insisting upon its fulfilment, would be following a mischievous and mistaken course; and that unless it be found that central action is required to make local action efficient, there is no reason for keeping up its machinery and employing its functionaries." (pp. 480.)

*Prolongation of Life in the Eighteenth Century.*—Dr. Southwood Smith has an admirable paper on this topic. One or two passages are worthy of transcription into every periodical:—

"It is a matter of familiar history that in this country the spirit of improvement which had been awakened in the two preceding centuries, exerted itself with extraordinary activity during the whole of the eighteenth century. Already forests had been cleared, marshes and swamps had been drained, and from the more settled government of the country, cities and towns, being no longer fortresses, had extended beyond the walls of their fortifications. But precisely at this period special attention also began to be paid to the well ordering, cleansing, and paving of towns. The narrow streets were widened, great numbers of houses were entirely taken down and rebuilt, slate being substituted for thatch and brick for timber, while the manufacture of glass was so much increased that glass windows, even in the poorer houses, became common. The general result was to render the houses more substantial and commodious, and the rooms larger, higher, lighter, warmer, and better ventilated. Agriculture took a surprising start, multiplying a hundredfold the production of fresh vegetable food, and increasing, perhaps, in a still more remarkable degree, the amount of fresh animal food, by the extension of the comparatively new art of collecting, storing, and preserving fodder for cattle in winter. The increased and successful activity of manufactures gave improved and cheap clothing to the people—clothing not only conducive to health through warmth, but almost equally so through cleanliness, for the articles of clothing now began to be composed of such tissues and textures as favour and compel frequent washing. At the same time disease in general assumed a milder form, and epidemics in particular became less frequent and far less formidable in their character.

"The combined operation of these causes goes far to account for so wonderful a change in the sanitary condition of the country as must have taken place before any considerable portion of the community could have obtained a lengthened life, equivalent to a fourth of its whole term.

"It is true these sanitary changes were effected without any knowledge of their sanitary nature or tendency, but they were not the less sanitary in their operation because that operation was undesigned and uncontemplated, nor because the changes in question were the pure result of the force of the onward current of civilization. . . . . .

"If the advancing civilization of the eighteenth century was accompanied by such a prolongation of life as we have seen was gained, what has been gained in the half century that followed—in the time in which we ourselves live? We cannot tell, because we have no record of definite facts to inform us. We know, indeed, that every sanitary agency that could have been in operation in the eighteeeth century has acquired greater energy in the nineteenth; that several powerful sanitary influences have been superadded, and that the main conditions on which life and health depend have recently experienced an expansion and improvement to which no former age presents a parallel." (Pp. 502-3.)

We must conclude with these few brief extracts. We regret it much. But the regret is softened by the fact that both the "Transactions" and membership of the Social Science Association, are open to all earnest men, anxious for the welfare of their kind, and the honour of their country.

